# In-process real-time probiotic phenotypic strain identity tracking: The use of Fourier transform infrared spectroscopy

**DOI:** 10.3389/fmicb.2022.1052420

**Published:** 2022-12-08

**Authors:** Francesca Deidda, Miriam Cordovana, Nicole Bozzi Cionci, Teresa Graziano, Diana Di Gioia, Marco Pane

**Affiliations:** ^1^Probiotical Research S.r.L, Novara, Italy; ^2^Bruker Daltonics GmbH & Co., Bremen, Germany; ^3^Department of Agricultural and Food Sciences, University of Bologna, Bologna, Italy

**Keywords:** FTIR spectroscopy, *Lactiplantibacillus plantarum*, probiotics, large-scale production, strain typing, IR Biotyper^®^

## Abstract

Probiotic bacteria, capable of conferring benefits to the host, can present challenges in design, development, scale-up, manufacturing, commercialization, and life cycle management. Strain identification is one of the main quality parameters; nevertheless, this task can be challenging since established methodologies can lack resolution at the strain level for some microorganisms and\or are labor-intensive and time-consuming. Fourier transform infrared spectroscopy (FTIRS) has been largely used for the investigation of pathogenic species in the clinical field, whereas only recently has been proposed for the identification of probiotic strains. Within the probiotic industrial production, bacterial strains can be subjected to stressful conditions that may affect genomic and phenotypic characteristics; therefore, real-time monitoring of all the sequential growth steps is requested. Considering the fast, low-cost, and high-throughput features, FTIRS is an innovative and functional technology for typing probiotic strains from bench-top experiments to large-scale industrial production, allowing the monitoring of stability and identity of probiotic strains. In this study, the discriminatory power of FTIRS was assessed for four *Lactiplantibacillus plantarum* probiotic strains grown under different conditions, including temperatures (30 and 37°C) and medium (broth and agar), after consecutive sub-culturing steps. A comparison between the generated spectra with pulsed-field gel electrophoresis (PFGE) profiles was also performed. FTIRS was not only able to distinguish the strains of *L. plantarum* under different growth conditions but also to prove the phenotypic stability of *L. plantarum* type strain LP-CT after six growing steps. Regardless of the growth conditions, FTIRS spectra related to LP-CT constituted a unique hierarchical cluster, separated from the other *L. plantarum* strains. These results were confirmed by a PFGE analysis. In addition, based on FTIRS data, broth cultures demonstrated a higher reproducibility and discriminatory power with respect to agar ones. These results support the introduction of FTIRS in the probiotic industry, allowing for the step-by-step monitoring of massive microbial production while also guaranteeing the stability and purity of the probiotic strain. The proposed novel approach can constitute an impressive improvement in the probiotic manufacturing process.

## Introduction

Fourier transform infrared spectroscopy (FTIRS), a technology traditionally used in the chemistry field for the qualitative and quantitative analysis of complex organic mixtures, has found important applications also in the microbiology field for microbial typing. The methodology is based on the absorption spectrum detected by FTIRS from bacterial cells, representing the bacteria’s specific fingerprint signature and reflecting its biomolecular content in relation to its genetic information (Helm et al., 1991; Naumann et al., 1991). Specifically, this methodology has been largely used for typing bacteria at different taxonomic levels, such as genera, species, and even at the strain level, especially human pathogens in the clinical field and for real-time outbreak investigation (Cordovana et al., 2021; Lombardo et al., 2021). The most common FTIRS-based system applied in clinical microbiology is the IR Biotyper^®^ commercialized by Bruker since, 2018 (Bruker GmbH Daltonics Division, 2018; Burckhardt et al., 2019; Martak et al., 2019; Hu et al., 2021).

The efficacy of FTIRS in identifying bacteria in comparison with DNA-based techniques, such as pulsed-field gel electrophoresis (PFGE), the more recent multilocus sequence typing (MLST), and the new gold standard whole-genome sequencing (WGS), has been largely demonstrated (Martak et al., 2019; Deidda et al., 2021; Hu et al., 2021). Although WGS remains the most powerful approach used to characterize strains accurately and interpret functions of LAB at the genome level (Buron-Moles et al., 2019; Sharma et al., 2020), it is hard to apply for routine analysis. In contrast, FTIRS can be highly attractive for probiotic industries because of its high-performance speed, low cost, and simplicity (Rodrigues et al., 2020; Song et al., 2020; Hu et al., 2021). The principle of FTIRS consists in measuring the absorption of infrared light by cell components, such as lipopolysaccharides, lipids, carbohydrates, and proteins, resulting in a specific FTIRS spectrum reflecting the overall composition of the sample and corresponding to a specific fingerprint signature (Naumann et al., 1991; Lasch and Naumann, 2015).

The first promising result has been demonstrated by Deidda et al. (2021). The study showed the success of this technology in typing at species, subspecies, and strain-level bacteria belonging to the *Bifidobacterium* genus, providing equivalent and also more informative outcomes with respect to genetic approaches, such as PFGE, MLST, and OrthoANI analysis. In particular, FTIRS technology showed a higher discriminatory power in distinguishing strains belonging to *Bifidobacterium longum* subsp. *longum* and *Bifidobacterium animalis* subsp. *lactis*, demonstrating to overcome some limitations of genomic-based methodologies and detecting phenotypic information that cannot be evidenced by DNA-based techniques. In addition, another work on discriminating *Lactiplantibacillus plantarum* strains demonstrated that FTIRS was not only comparable to WGS and PFGE but also had a higher discriminatory power than MLST (Li et al., 2022). This complies with the fact that the identification of bacteria at the strain level, revealing genetic information evidenced by phenotypic characteristics, is becoming increasingly important in modern microbiology.

According to Li et al. (2022), during microorganisms’ large-scale production, considering the intraspecific variation, different modes of action, and required manufacturing and quality control processes, strain-specific discrimination is essential to guarantee stability, quality, safety, and efficacy of probiotics. Stage et al. (2020) elegantly demonstrated the genomic and phenotypic stability of *Lactiplantibacillus rhamnosus* GG using a combined WGS and phenotypic *in vitro* testing. However, laboratory-scale genomic studies have important limitations not only due to the impossibility to run real-time monitoring of the process and its related costs but also considering the objective complexity of the interpretation of the results. In this scenario, FTIRS can play a key role in the characterization after strain isolation, discrimination, and cultivation within probiotic industrial production. Specifically, in a large-scale industrial production, bacteria may be subjected to certain stressful conditions, such as acid stress, nutrient deprivation, and temperature shift shock during a fermentative process that may affect genomic stability but most importantly phenotypic characteristics. We speculate that FTIRS can constitute a valid and advantageous strategy for monitoring the phenotype stability of probiotic strains in a large-scale production process in real-time.

Among homofermentative lactic acid bacterium (LAB), *L. plantarum* has been widely used as a model species for metabolic, ecological, and genetic studies, having commercial importance as a starter culture for multiple food fermentations and as probiotic culture (Zheng J. et al., 2020). This species has been included in the list of microorganisms with qualified presumption of safety (QPS) (EFSA Panel on Biological Hazards (Biohaz) et al., 2018) and strains of *L. plantarum* have been shown to exhibit anti-inflammatory activities against inflammation-related diseases, especially inflammatory bowel diseases (IBDs) (Liu et al., 2018).

Considering its broad environmental and host colonization, such as human breast milk, human feces, cheese, and fermented food products (Liu et al., 2018), *L. plantarum* strains showed significant intraspecific genetic and phenotypic variability due to strain-specific genes (Salvetti et al., 2018; Cen et al., 2020; Pan et al., 2021); these features represent an interesting challenge to be studied and monitored with FTIRS.

The aim of this study was first to confirm that FTIRS can discriminate *L. plantarum* strains belonging to probiotical strain collection and second to monitor in real-time the phenotypic stability of a targeted strain of *L. plantarum* during consecutive propagation steps at different temperatures under laboratory conditions. A comparison between the generated spectra and PFGE profiles was also performed. Moreover, different growth conditions were considered: (i) temperature: 30 and 37°C for 18 h and (ii) medium: broth, which allowed testing on a heterogeneous bacterial population, and agar media, which allowed testing on a monoclonal colony.

## Materials and methods

### Bacterial cultures, growth conditions, and sample preparation

**FIGURE 1:**
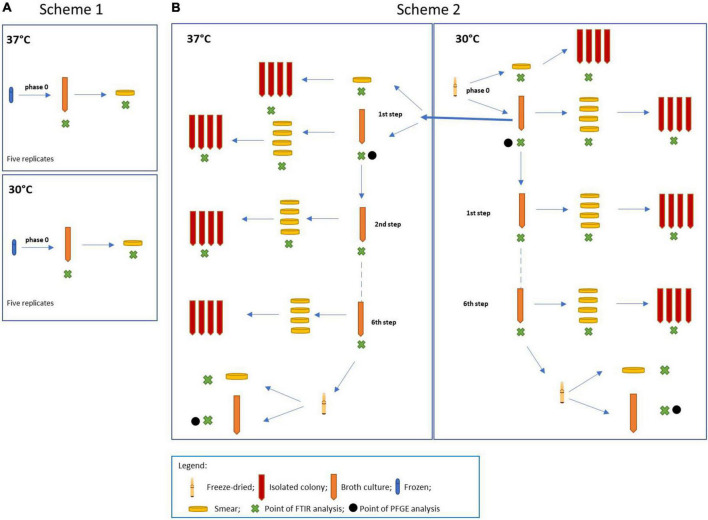
**(A)** Scheme 1: experimental scheme for the control strains *Lactiplantibacillus plantarum* LP01, LP02, and LP09; **(B)** scheme 2: experimental scheme for the type strain *L. plantarum* DSM 20174 (LP–CT).

For the experiment, four different *L. plantarum* strains were used, such as *L. plantarum* DSM 20174 type strain from the DSMZ culture collection (LP-CT in this study), *L. plantarum* LMG P-21021 (LP01 in this study), *L. plantarum* LMG P-21020 (LP02 in this study), and *L. plantarum* DSM 25710 (LP09 in this study), from the collection belonging to the Probiotical Spa company. The experimental design is shown in Figure 1. Two procedures were carried out at the same time: scheme 1 including the three strains of *L. plantarum* (LP01, LP02, and LP09), used as the control group (Figure 1A); scheme 2 regarding the type strain LP-CT (Figure 1B).

The original lyophilized LP-CT strain was resuspended in 1 ml of MRS broth (BD Difco, Becton Dickinson, Italy) (“phase 0”). After an acclimatization time of 15 min, it was streaked on MRS agar (BD Difco) and inoculated at 2% in MRS broth. The broth phase samples were incubated at 30°C for 18 h, while the agar phase samples were at 30°C for 48 h. From “phase 0” cultures, four different growth and incubation conditions were prepared: broth 30°C/18 h by inoculation at 2%, agar 30°C/48 h by smear, broth 37°C 18 h by inoculation at 2%, and agar 37°C/48 h by smear. From each smear, single-colony sub-cultures were carried out in broth. From each broth culture, four smears on agar plates were made. The LP-CT underwent six sequential transplants and six re-isolations under four growth conditions (scheme 2, Figure 1B).

The other three strains (LP01, LP02, and LP09), starting from a frozen culture at −80°C, were revitalized five times in MRS broth (phase 0) under the same conditions as LP-CT (30 and 37°C for 18 h).

Each culture (in both agar and liquid mediums) underwent IR Biotyper analysis (Bruker Daltonics GmbH & Co. KG), following the manufacturer’s instructions for sample preparation and spectra acquisition. For the agar plate culture, bacterial biomass (a full 1 μl loop) was resuspended in 50 μl of 70% ethanol solution. After vortexing until complete resuspension, 50 μl of sterile water was added. For the broth cultures, 5 ml of culture was centrifuged at 5,000 rpm for 5 min. After discarding the supernatant, the cells were washed two times using 5 ml of sterile Dulbecco’s phosphate-buffered saline buffer 1× (DPBS) and 5 min of centrifugation at 5,000 rpm. The washed cells were then resuspended in 500 μl of DPBS buffer and used for IR Biotyper measurement. For all the samples, 15 μl of bacterial suspension were spotted in quadruplicate onto the IR Biotyper 96-wells silicon sample plate. The plate was then left at room temperature until the spots were completely dry (ca. 30–40 min) and then subjected to infrared spectroscopy analysis. “Phase 0” was then subjected to transplants (scheme 1, Figure 1A).

### Pulsed-field gel electrophoresis analysis

LP-CT cultures at the beginning and at the end of the process from scheme 2 were subjected to PFGE analysis. Four cultures were involved: phase 0 at 30°C, first step at 37°C, freeze-dried at 30°C, and 37°C (Figure 1B). The protocol was described by Deidda et al. (2021).

### Spectra acquisition by IR Biotyper^®^ and data analysis

Spectra acquisition was performed using the IR Biotyper^®^ system (Bruker Daltonics) and the OPUS software (Bruker Optics GmbH & Co. KG, Germany) in transmission mode in the spectral range of 4,000–5,00 cm-1 (mid-IR). Spectra processing and visualization were performed with the IR Biotyper^®^ Client software V3.1 and V4.0 (Bruker Daltonics) and using default settings recommended by the manufacturer. After spectra smoothing using the Savitzky–Golay algorithm over nine data points, the second derivative of the spectra was calculated. Spectra were then cut to 1,300–800 cm^–1^ (Baker et al., 2014) and vector-normalized, to correct for the preparation-related variance of biomass and hence absorption. For each run, quality control was performed with the Infrared Test Standards (IRTS 1 and 2) in the IR Biotyper^®^ kit. All spectra were acquired by intercalating a background spectrum between each sample/control measurement.

Hierarchical cluster analysis (HCA) and linear discriminant analysis (LDA) were applied for unsupervised and supervised multivariate analyses, using the IR Biotyper^®^ V3.1 software functionality. Euclidean and Pearson’s correlation were used as the clustering method, and single linkage, average linkage, complete linkage, and Ward’s were used as linkage types. The spectral distance, variance, and clustering cut-off values were analyzed, and the reproducibility of the experiment and the discriminatory power of IR Biotyper^®^ were evaluated for all four incubation protocols by calculating the distance between all the spectra of each isolate (intra-isolate Euclidean distance) and the difference between the intra-isolate distance and the inter-isolates distance following the manufacturer’s instruction. The normality and homogeneity of variance of Euclidean distances among isolates were checked; statistical significance was evaluated with one-way ANOVA or Kruskal test with Bonferroni correction in R software (version 3.5.3). HCA was carried out on both schemes 1 and 2 to investigate the stability of LP-CT over the time, analyzing the strain after six serial passages in culture.

## Results

### Scheme 1: Control strains vs. type strain LP-CT

**FIGURE 2:**
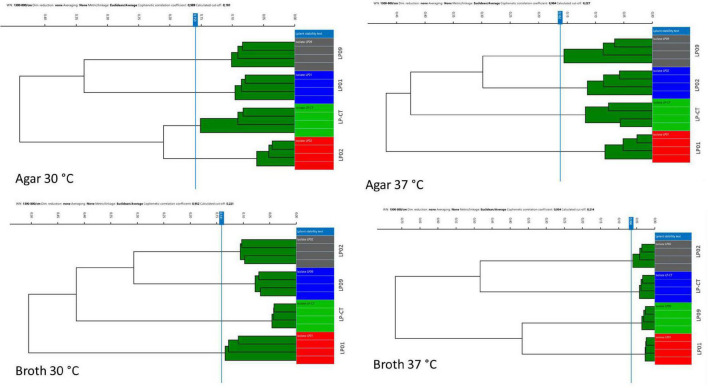
Hierarchical cluster analysis (HCA) results for the four incubation protocols from experimental scheme 1. Each isolate is represented by its four technical replicates (on the right side of each image), and it is depicted with a different color. On the left side of each image, the IR Biotyper clustering of the spectra into the corresponding isolates is shown, as well as the relationship between the different isolates (indicated by the branches of the dendrogram).

All the different clustering methods evaluated relying on different metrics (Euclidean and Pearson’s correlation) and different linkage types (single linkage, average linkage, complete linkage, and Ward’s) showed very similar results (data not shown). The method which showed the clearest visualization of results (Euclidean average linkage) was selected to create the figures. Overall, HCA showed that all four incubation protocols enabled the clear separation of the four isolates, LP01, LP02, LP09, and LP-CT (cut-off values: 0.159 agar 30°C, 0.162 agar 37°C, 0.141 broth 30°C, and 0.066 broth 37°C) (Figure 2).

Moreover, the samples prepared in broth (both at 30 and 37°C) showed a lower variance with respect to the agar ones, evidenced by the reduced SD of intra-isolate distance averages (Table 1). The values of intra-isolate and inter-isolate distances were significantly different considering all the experimental conditions, with the exception of agar at 37°C and broth at 37°C. The lowest intra-isolate variance was the preparation broth at 37°C, resulting in the highest reproducibility condition. The same condition resulted to have the most resolution power, showing a high value of difference intra-/inter-isolate distance (Table 1).

**TABLE 1:** Assessment of the validity of IR Biotyper^®^ results for scheme 1.

Growth condition	Intra-isolate distance (Euclidean distance)	Inter-isolate distance (Euclidean distance)	Difference intra-/inter-isolate distance
Agar 30°C	0.07 ± 0.033^a^	0.52 ± 0.035^a^	0.45
Agar 37°C	0.09 ± 0.032^b^	0.43 ± 0.016^b^	0.34
Broth 30°C	0.09 ± 0.006^b^	0.47 ± 0.005^b^	0.38
Broth 37°C	0.03 ± 0.006^c^	0.62 ± 0.005^c^	0.59

For each incubation protocol, the distance between all the spectra of each isolate (intra-isolate distance) and the distance between each isolate and the other ones (inter-isolates distance) were calculated; the values are expressed as the average of absolute values ± standard deviation. Different lower-case letters indicate significant differences (*p*-value < 0.05).

### Schemes 1 and 2: Control strains vs. multiple growth passages of type strain LP-CT

**FIGURE 3:**
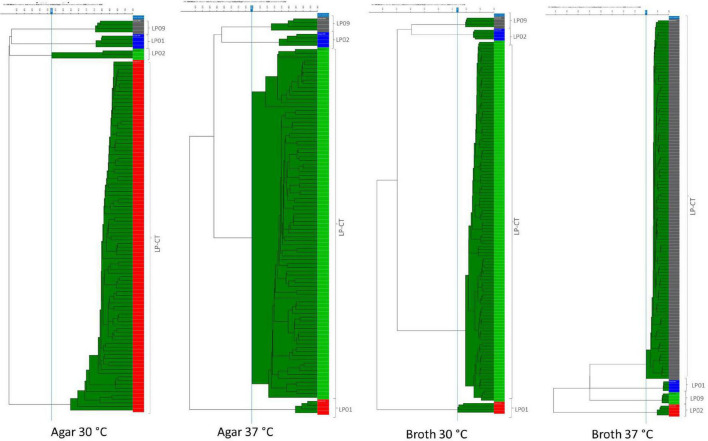
HCA results for the four incubation protocols from scheme 2 and scheme 1. LP01, LP02, LP09 are represented with different colors by their four technical replicates (on the right side of each image); LP-CT is represented by all its technical and biological replicates. On the left side of each image, the IR Biotyper clustering of the spectra into the corresponding isolates is shown, as well as the relationship between the different isolates (indicated by the branches of the dendrogram).

The addition of spectra measured from the six sub-culturing steps of LP-CT of experimental scheme 2 to experimental scheme 1 did not influence the IR Biotyper clustering. Considering all the cultivation conditions for all strains, the isolates derived from each strain clustered separately (Supplementary Figure 1). All isolates of LP-CT derived from the sub-cultures constituted a unique cluster, separated from the other strains LP01, LP02, and LP09, under all the growth conditions experimented (cut-off values: 0.209 agar 30°C, 0.183 agar 37°C, 0.131 broth 30°C, and 0.100 broth 37°C) (Figure 3).

In comparison to the dataset with only one step (scheme 1), the presence of six serial sub-culturing steps of LP-CT resulted in a higher inter-isolate variance, a lower intra-isolate distance, and a difference intra-/inter-isolate distance.

As observed in the previous step, broth cultures showed a better performance in comparison with the agar ones (Table 2). Specifically, broth culture at 30°C showed a significantly lower intra-isolate distance and, therefore, a higher reproducibility with respect to the other conditions. Broth cultures at both the experimental temperatures exhibited higher values of intra-/inter-isolate distance, especially for 37°C, indicating an elevated discriminating power of IR Biotyper^®^.

**TABLE 2:** Assessment of the validity of IR Biotyper^®^ results for schemes 1 and 2.

Growth condition	Intra-isolate distance (Euclidean distance)	Inter-isolate distance (Euclidean distance)	Difference intra-/inter-isolate distance
Agar 30°C	0.17 ± 0.003^a^	0.45 ± 0.029^a^	0.28
Agar 37°C	0.24 ± 0.032^b^	0.43 ± 0.031^b^	0.19
Broth 30°C	0.15 ± 0.006^c^	0.47 ± 0.018^c^	0.31
Broth 37°C	0.17 ± 0.006^a^	0.54 ± 0.042^d^	0.37

For each incubation protocol, the distance between all the spectra of each isolate (intra-isolate distance) and the distance between each isolate and the other ones (inter-isolates distance) were calculated; the values are expressed as the average of absolute values ± standard deviation. Different lower-case letters indicate significant differences (*p*-value < 0.05).

**FIGURE 4:**
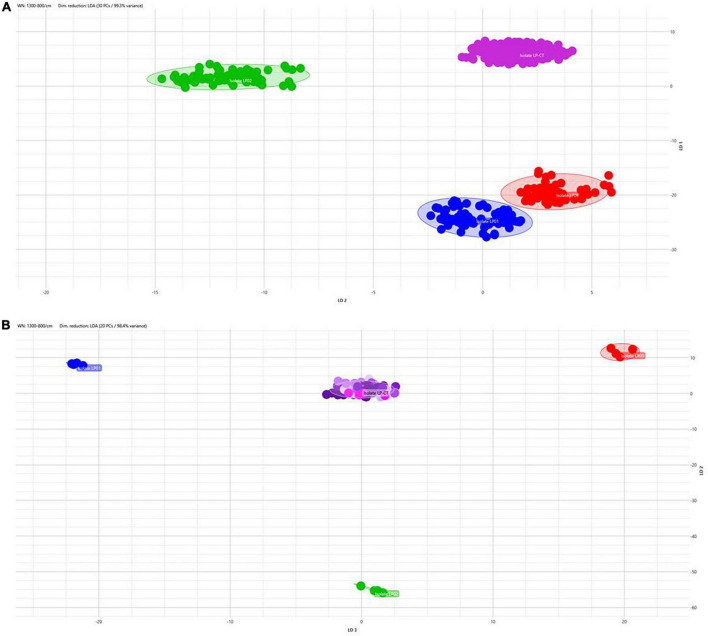
**(A)** 2D scatterplot of all the spectra included in this study, including all the isolates and all the incubation protocols (scheme 2) and applying LDA analysis with 30 PCs (representing 99.3% of variance). Isolate LP-CT is depicted in violet, LP01 in blue, LP02 in green, and LP09 in red. Each geometric form represents an IRBT spectrum. **(B)** 2D scatterplot displaying the four isolates in the preparation broth 37°C (LP-CT with all its passages). Each isolate is represented by different colors (LP-CT in violet, LP01 in blue, LP02 in green, and LP09 in red). Different shades of color represent the culture steps, in a gradient from the oldest (lighter) to the newest (darker), and the final lyophilized sample is displayed in magenta. The scatterplot was created using LDA with 20 principal components (PCs), corresponding to 98.4% of variance.

The application of LDA enabled nullifying the medium- and incubation-related variance and achieving a clear separation of the isolates also analyzing together all the spectra derived from all the incubation conditions (Figure 4A). All the isolates are clearly distinguished from the others. Figure 4B shows LDA investigation on the stability of the strain over the subsequent steps in culture. For the control strains LP01, LP02, and LP09, all the steps on broth medium at 37°C are reported; for LP-CT, all the passages within scheme 2 are presented. Particularly, for LP-CT, the different shades of color represent the acquisition date and, therefore, the culture steps, in a gradient from the oldest (lighter) to the newest (darker). The LP-CT final lyophilized sample is displayed in magenta. The halo corresponds to the 0.95 confidence interval. Also, in this analysis, no drift of spectra could be observed over time and the discrimination between LP-CT and the other isolates appeared clear and undoubtful.

### Pulsed-field gel electrophoresis on scheme 2: *Lactiplantibacillus plantarum* LP-CT profiles

**FIGURE 5:**
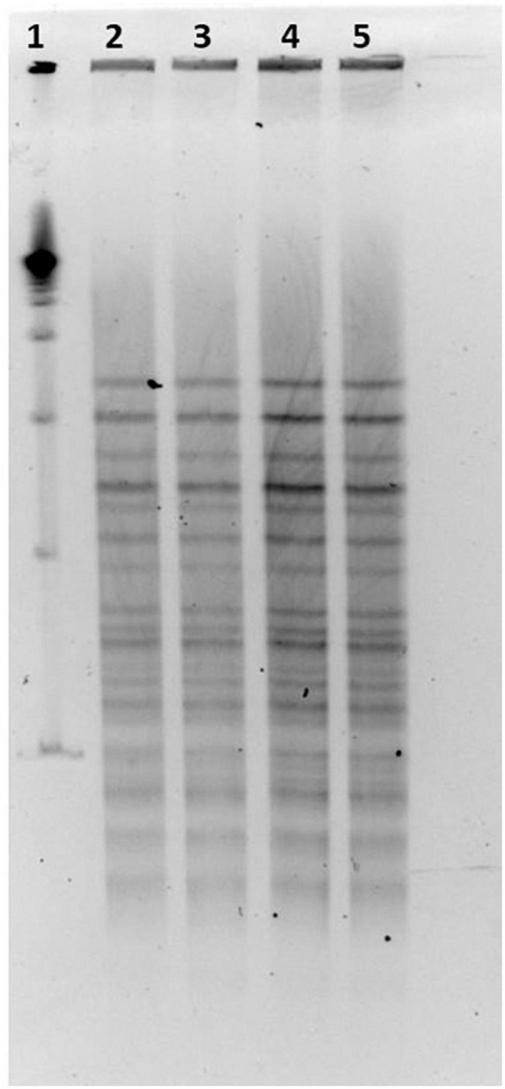
(1) LP-CT strain PFGE profiles with *Not*I; 19 marker; (2) LP-CT 1° step at 30°C; (3) LP-CT freeze-dried at 30°C; (4) LP-CT 1° step at 37°C; and (5) LP-CT freeze-dried at 37°C.

LP-CT strains deriving from broth and agar cultures at 30°C from phase 0 and from first step at 37°C, and freeze-dried at 30 and 37°C, showed the same PFGE profiles, and the patterns were not discernible (Figure 5).

## Discussion

In this study, the discriminatory power of the FTIRS-based IR Biotyper^®^ system in typing probiotic bacteria has been assessed using strains belonging to *L. plantarum*, a common probiotic species used both in humans and animals (Zheng Z. Y. et al., 2020).

In the last decade, the use of this technique for typing probiotic bacteria has gained a huge interest in the field of clinical microbiology, and, more recently, in the field of probiotic investigation. A pioneering study was carried out by our research group, involving *B. longum* subsp. *longum* and *B. animalis* subsp. *lactis* strains and showing the power of FTIRS in discriminating bifidobacteria at species, subspecies, and even at strain level (Deidda et al., 2021). The FTIRS technology has been proven to distinguish only at species level members belonging to *L. plantarum* (Oust et al., 2004; Bosch et al., 2006; Santos et al., 2015; Quintelas et al., 2018). A very recent work (Li et al., 2022) evidenced the strain-typing power of this technique in *L. plantarum*, whose results are consistent with those reported in the current work.

After the confirmation of the ability to distinguish the strains of *L. plantarum* involved in this study (LP01, LP02, LP09, and LP-CT), using different growth conditions (broth and agar cultures, 30 and 37°C as incubation temperatures), FTIRS was used to prove the phenotypic stability of *L. plantarum*-type strain LP-CT after six sub-culturing steps and freeze-drying steps, simulating the number of bacterial replication of a probiotic industrial process. Considering broth cultures, agar smears, and isolated colonies at 30 and 37°C, the IR spectra related to LP-CT formed a unique hierarchical cluster, maintaining its separation from LP01, LP02, and LP09. LDA analysis reported the same grouping trend, with a clear differentiation of each strain independently on the adopted growing condition. IR Biotyper^®^ results were validated at the beginning steps and at the final steps by a PFGE analysis.

Microbial evolution studies demonstrated that nutrient deficiency and stressful conditions can contribute to genotypic and phenotypic alterations of strains (Saxelin et al., 1999; Péter and Reichart, 2001; Saarela et al., 2004; Douillard et al., 2016). Culturing in an industrial process has very different strain requirements than laboratory culturing, especially in terms of costs, time, and yield. Each step in the process depends on the prior step and it is important to identify strain-dependent sensitivities, preserving the overall health of the cells while proceeding through the process (Fenster et al., 2019). Some obstacles, such as acid stress and depletion of nutrients, that may occur during industrial production with batch fermentation may lead to increased DNA mutation and recombination rates (Stage et al., 2020). It is, therefore, necessary to adopt highly settled strategies to obtain maximum yield in minimal time and to guarantee the stability of microbial production. It is necessary to point out that the experiments carried out in the current work were set to mimic the number of bacterial replications in an industrial process, in order to verify possible phenotypic drifts to the detriment of the whole process. An objective limitation of the present experiment was the limited number of *L. plantarum* strains available for the strain discrimination challenge with FTIRS and the over-simplified fermentation protocol since it was not possible to simulate exactly all the stressful events that can occur during an industrial production (e.g., high-sheer stress, neutralizers, prolonged operation in upstream and downstream process, biomass separation, and freeze-drying).

Our experiment confirmed the possibility suggested by our previous work (Deidda et al., 2021) to apply the IR Biotyper^®^ as a quick, inexpensive, and high-throughput tool for strain tracking during an industrial production process. In concomitance with measures taken to optimize fast growth and high survival rates, FTIRS analysis can allow checking the authenticity of a probiotic strain during the large-scale production process. This experiment demonstrated that it was possible to obtain phenotypic discrimination by FTIRS within an industrial probiotic production, which is equivalent to genotypic discrimination, such as PFGE (Deidda et al., 2021).

Even though DNA-based techniques were successful in typing *L. plantarum* at interspecies and intraspecies levels (Evanovich et al., 2019; Manzoor and Tayyeb, 2019; Yu et al., 2021), some phenotypic variations associated with the cell surface can be eluded. In this context, it is necessary to highlight the cell membrane-associated capability of *L. plantarum* of resisting acid, heat, and other stresses (Capozzi et al., 2011; Ricciardi et al., 2012). Based on these information and properties, a technology like FTIRS can detect modifications in the bacterial external surfaces, which are not always ascribed to genetic differences, and can pave the way to an identification process based purely on phenotypic features.

Moreover, FTIRS proved to be effective in distinguishing at strain-level probiotic bacteria deriving from different growth conditions. This promising aspect of technology, together with the already explained advantages and the possibility to construct well-composed IR Biotyper^®^ databases, can lead to the development of an “artificial intelligence” tool able to recognize probiotic strains despite variability, growth conditions, and source of isolation, helping also in setting up experiments with high reproducibility. Therefore, the introduction of this technology may contribute to fast and standardized strain identification since sometimes findings are not paired with a definitive identification procedure, leaving the pertinence of the results mostly to the laboratory’s good practices.

Within a large-scale production, it is possible to obtain a potential routine control process with the insertion of FTIRS analysis in each cultivation step, as demonstrated in the experimental scheme 2 as an upstream monitoring, whereas DNA-based analysis can be used only as a downstream and/or upstream check. Given the advantages of ease of execution and user-friendless with respect to DNA-based techniques, a step-by-step control by FTIRS can allow for the simple and fast identification of a possible contamination/alteration of the original culture.

An additional positive result is the possibility of using a broth culture, instead of the traditional smear from the agar plate. The higher discriminating power using broth culture, with respect to the other conditions can be justified by: (i) a possible reduction of experimental interferences due to the collection of agar particles and old cells and (ii) the presence of a more heterogeneous and fresh bacterial population in broth with respect to agar medium.

For these reasons, the introduction of FTIRS in the probiotic industry can constitute a positive implementation, considering the possibility of ensuring the stability and the identity of the starting strain and obtaining step-by-step monitoring of the probiotic culture.

## Conclusion

Reproducibility, high performance, and quality are important requirements in industrial manufacturing probiotic processes. This study confirmed the capability of FTIRS in discriminating *L. plantarum* strains under different laboratory growth conditions and demonstrated FTIRS efficacy in ensuring the phenotypic stability, identity, and purity of *L. plantarum* type strain LP-CT during consecutive propagation steps. Considering the already proven discriminatory power in probiotic bacteria, as well as its quick, inexpensive, and high-throughput tools, FTIRS can constitute a suitable methodology for routine application in microbial production within the probiotic industry, guaranteeing both in-process strain identity and phenotypic monitoring.

## Data availability statement

The original contributions presented in this study are included in the article/Supplementary material, further inquiries can be directed to the corresponding author.

## Author contributions

MP, FD, and MC conceived the study. FD and TG performed the experiments. MC performed the data analysis. NB interpreted the results and contributed to the writing of the manuscript. DD and MP critically revised the final manuscript. All authors contributed to the article and approved the submitted version.

## References

[B1] Baker M. J. Trevisan J. Bassan P. Bhargava R. Butler H. J. Dorling K. M. (2014). Using Fourier transform IR spectroscopy to analyze biological materials. *Nat. Protoc.* 9 1771–1791. 10.1038/nprot.2014.110 24992094 PMC4480339

[B2] Bosch A. Golowczyc M. A. Abraham A. G. Garrote G. L. De Antoni G. L. Yantorno O. (2006). Rapid discrimination of lactobacilli isolated from kefir grains by FT-IR spectroscopy. *Int. J. Food Microbiol*. 111 280–287. 10.1016/j.ijfoodmicro.2006.05.010 16860422

[B3] Bruker GmbH Daltonics Division (2018). *Bruker GmbH Announces Improved Solutions for Microbial Strain Typing, Hospital Hygiene and Infection control, and Candida Auris Testing at ASM 2018.* Available online at: https://ir.bruker.com/press-releases/press-release-details/2018/Bruker-Announces-Improved-Solutions-for-Microbial-Strain-Typing-Hospital-Hygiene-and-Infection-Control-and-Candida-auris-Testing-at-ASM-2018/default.aspx (accessed on June 21, 2022).

[B4] Burckhardt I. Sebastian K. Mauder N. Kostrzewa M. Burckhardt F. Zimmermann S. (2019). Analysis of Streptococcus pneumoniae using Fouriertransformed infrared spectroscopy allows prediction of capsular serotype. *Eur. J. Clin. Microbiol. Infect. Dis.* 38 1883–1890. 10.1007/s10096-019-03622-y 31286288 PMC6778537

[B5] Buron-Moles G. Chailyan A. Dolejs I. Forster J. Mikš M. H. (2019). Uncovering carbohydrate metabolism through a genotype-phenotype association study of 56 lactic acid bacteria genomes. *Appl. Microbiol. Biotechnol.* 103 3135–3152. 10.1007/s00253-019-09701-6 30830251 PMC6447522

[B6] Capozzi V. Weidmann S. Fiocco D. Rieu A. Hols P. Guzzo J. (2011). Inactivation of a small heat shock protein affects cell morphology and membrane fluidity in *Lactobacillus plantarum* WCFS1. *Res. Microbiol.* 162 419–425. 10.1016/j.resmic.2011.02.010 21349328

[B7] Cen S. Yin R. Mao B. Zhao J. Zhang H. Zhai Q. (2020). Comparative genomics shows niche-specific variations of *Lactobacillus plantarum* strains isolated from human, *Drosophila melanogaster*, vegetable and dairy sources. *Food Biosci*. 35:100581. 10.1016/j.fbio.2020.100581

[B8] Cordovana M. Mauder N. Kostrzewa M. Wille A. Rojak S. Hagen R. M. (2021). Classification of *Salmonella enterica* of the (Para-)Typhoid Fever Group by Fourier-Transform Infrared (FTIR) Spectroscopy. *Microorganisms* 9:853. 10.3390/microorganisms9040853 33921159 PMC8071548

[B9] Deidda F. Bozzi Cionci N. Cordovana M. Campedelli I. Fracchetti F. Di Gioia D. (2021). Bifidobacteria strain typing by fourier transform infrared spectroscopy. *Front. Microbiol.* 12:692975. 10.3389/fmicb.2021.692975 34589064 PMC8473902

[B10] Douillard F. P. Ribbera A. Xiao K. Ritari J. Rasinkangas P. Paulin L. (2016). Polymorphisms, chromosomal rearrangements, and mutator phenotype development during experimental evolution of *Lactobacillus rhamnosus* GG. *Appl. Environ. Microbiol.* 82 3783–3792. 10.1128/AEM.00255-16 27084020 PMC4907198

[B11] EFSA Panel on Biological Hazards (Biohaz) Ricci A. Allende A. Bolton D. Chemaly M. Davies R. (2018). Update of the list of QPS-recommended biological agents intentionally added to food or feed as notified to EFSA 7: Suitability of taxonomic units notified to EFSA until September 2017. *EFSA J.* 16:5131. 10.2903/j.efsa.2018.5131 32625678 PMC7328878

[B12] Evanovich E. De Souza Mendonça Mattos P. J. Guerreiro J. F. (2019). Comparative genomic analysis of *Lactobacillus plantarum*: An overview. *Int. J. Genomics* 2019:4973214. 10.1155/2019/4973214 31093491 PMC6481158

[B13] Fenster K. Freeburg B. Hollard C. Wong C. Rønhave Laursen R. Ouwehand A. C. (2019). The production and delivery of probiotics: A review of a practical approach. *Microorganisms* 7:83. 10.3390/microorganisms7030083 30884906 PMC6463069

[B14] Helm D. Labischinski H. Schallehn G. Naumann D. (1991). Classification and identification of bacteria by Fourier-transform infrared spectroscopy. *J. Gen. Microbiol.* 137 69–79. 10.1099/00221287-137-1-69 1710644

[B15] Hu Y. Zhou H. Lu J. Sun Q. Liu C. Zeng Y. (2021). Evaluation of the IR Biotyper for *Klebsiella pneumoniae* typing and its potentials in hospital hygiene management. *Microb. Biotechnol*. 14 1343–1352. 10.1111/1751-7915.13709 33205912 PMC8313285

[B16] Lasch P. Naumann D. (2015). “Infrared spectroscopy in microbiology,” in *Encyclopedia of analytical chemistry*, ed. Meyers R. A. (Chichester: John Wiley & Sons Ltd.), 1–32.

[B17] Li X. Zhu L. Wang X. Li J. Tang B. (2022). Evaluation of IR Biotyper for *Lactiplantibacillus plantarum* Typing and Its Application Potential in Probiotic Preliminary Screening. *Front. Microbiol*. 13:823120. 10.3389/fmicb.2022.82312 PMC8988154 35401469

[B18] Liu Y. W. Liong M. T. Tsai Y. C. (2018). New perspectives of *Lactobacillus plantarum* as a probiotic: The gut-heart-brain axis. *J. Microbiol.* 56 601–613. 10.1007/s12275-018-8079-2 30141154

[B19] Lombardo D. Cordovana M. Deidda F. Pane M. Ambretti S. (2021). Application of Fourier transform infrared spectroscopy for real-time typing of *Acinetobacter baumannii* outbreak in intensive care unit. *Future Microbiol.* 16 1239–1250. 10.2217/fmb-2020-0276 34674538

[B20] Manzoor A. Tayyeb A. (2019). Functional probiotic attributes and gene encoding plantaracin among variant *Lactobacillus Plantarum* strains. *Microb. Pathog*. 131 22–32. 10.1016/j.micpath.2019.03.016 30902731

[B21] Martak D. Valot B. Sauget M. Cholley P. Thouverez M. Bertrand X. (2019). Fourier-transform infrared spectroscopy can quickly type Gram negative bacilli responsible for hospital outbreaks. *Front. Microbiol.* 10:1440. 10.3389/fmicb.2019.01440 31293559 PMC6606786

[B22] Naumann D. Helm D. Labischinski H. (1991). Microbiological characterization by FT-IR spectroscopy. *Nature* 351 81–82. 10.1038/351081a0 1902911

[B23] Oust A. Møretrø T. Kirschner C. Narvhus J. A. Kohler A. (2004). Evaluation of the robustness of FT-IR spectra of lactobacilli towards changes in the bacterial growth conditions. *FEMS Microbiol. Lett.* 239 111–116. 10.1016/j.femsle.2004.08.024 15451108

[B24] Pan Q. Cen S. Yu L. Tian F. Zhao J. Zhang H. (2021). Niche-Specific adaptive evolution of *Lactobacillus plantarum* strains isolated from human feces and paocai. *Front. Cell. Infect. Microbiol*. 10:615876. 10.3389/fcimb.2020.615876 33489942 PMC7817898

[B25] Péter G. Reichart O. (2001). The effect of growth phase, cryoprotectants and freezing rates on the survival of selected micro-organisms during freezing and thawing. *Acta Aliment* 30 89–97. 10.1556/AAlim.30.2001.1.10

[B26] Quintelas C. Ferreira E. C. Lopes J. A. Sousa C. (2018). An overview of the evolution of infrared spectroscopy applied to bacterial typing. *Biotechnol. J.* 13:1700449. 10.1002/biot.2017004 29090857

[B27] Ricciardi A. Parente E. Guidone A. Ianniello R. G. Zotta T. Sayem S. M. A. (2012). Genotypic diversity of stress response in *Lactobacillus plantarum, Lactobacillus paraplantarum* and *Lactobacillus pentosus*. *Int. J. Food Microbiol.* 157 278–285. 10.1016/j.ijfoodmicro.2012.05.018 22704047

[B28] Rodrigues C. Sousa C. Lopes J. A. Novais Â Peixe L. (2020). A Front Line on *Klebsiella pneumoniae* Capsular Polysaccharid Knowledge: Fourier Transform Infrared Spectroscopy as an Accurate and Fast Typing Tool. *Msystems* 5 e00386–19. 10.1128/mSystems.00386-19 32209717 PMC7093823

[B29] Saarela M. Rantala M. Hallamaa K. Nohynek L. Virkajärvi I. Mättö J. (2004). Stationary-phase acid and heat treatments for improvement of the viability of probiotic lactobacilli and bifidobacteria. *J. Appl. Microbiol.* 96 1205–1214. 10.1111/j.1365-2672.2004.02286.x 15139911

[B30] Salvetti E. Harris H. M. B. Felis G. E. O’Toole P. W. (2018). Comparative genomics of the genus *Lactobacillus* reveals robust phylogroups that provide the basis for reclassification. *Appl. Environ. Microbiol.* 84 e00993–18. 10.1128/AEM.00993-18 29915113 PMC6102987

[B31] Santos M. I. Gerbino E. Tymczyszyn E. Gomez-Zavaglia A. (2015). Applications of infrared and raman spectroscopies to probiotic investigation. *Foods* 4 283–305. 10.3390/foods4030283 28231205 PMC5224548

[B32] Saxelin M. Grenov B. Svensson U. Fondén R. Reniero R. Mattila-Sandholm T. (1999). The technology of probiotics. *Trends Food Sci. Technol.* 10 387–392. 10.1016/S0924-2244(00)00027-3

[B33] Sharma A. Lee S. Park Y. S. (2020). Molecular typing tools for identifying and characterizing lactic acid bacteria: A review. *Food Sci. Biotechnol.* 29 1301–1318. 10.1007/s10068-020-00802-x 32995049 PMC7492335

[B34] Song J. Jongmans-Hochschulz E. Mauder N. Imirzalioglu C. Wichels A. Gerdts G. (2020). The Travelling Particles: Investigating microplastics as possible transport vectors for multidrug resistant *E. coli* in the Weser estuary (Germany). *Sci. Total Environ.* 720:137603. 10.1016/j.scitotenv.2020.137603 32143053

[B35] Stage M. Wichmann A. Jørgensen M. Vera-Jimenéz N. I. Wielje M. Nielsen D. S. (2020). *Lactobacillus rhamnosus* GG genomic and phenotypic stability in an industrial production process. *Appl. Environ. Microbiol.* 86:e02780-19. 10.1128/AEM.02780-19 31924618 PMC7054085

[B36] Yu A. O. Goldman E. A. Brooks J. T. Golomb B. L. Yim I. S. Gotcheva V. (2021). Strain diversity of plant-associated *Lactiplantibacillus plantarum*. *Microb. Biotechnol.* 14 1990–2008. 10.1111/1751-7915.13871 34171185 PMC8449665

[B37] Zheng J. Wittouck S. Salvetti E. Franz C. M. A. P. Harris H. M. B. Mattarelli P. (2020). A taxonomic note on the genus *Lactobacillus*: Description of 23 novel genera, emended description of the genus *Lactobacillus* beijerinck 1901, and union of *Lactobacillaceae* and *Leuconostocaceae*. *Int. J. Syst. Evol. Microbiol.* 70 2782–2858. 10.1099/ijsem.0.004107 32293557

[B38] Zheng Z. Y. Cao F. W. Wang W. J. Yu J. Chen C. Chen B. (2020). Probiotic characteristics of *Lactobacillus plantarum* E680 and its effect on Hypercholesterolemic mice. *BMC Microbiol.* 20:239. 10.1186/s12866-020-01922-4 32753060 PMC7401229

