# Supplementary material for: In-process real-time probiotic phenotypic strain identity tracking: The use of Fourier transform infrared spectroscopy
Source: Front Microbiol. 2022 Dec 8;13:1052420. doi: 10.3389/fmicb.2022.1052420 (PMC9772554; doi:10.3389/fmicb.2022.1052420)
Supplement: Supplementary file 1 [file Data_Sheet_1.zip › Table 1.DOCX]

Supplementary Material

## Supplementary Figures

**Supplementary Figure 1.** HCA results for all strains with all passages and all incubation protocols, performed using Euclidean average linkage. Each isolate is represented by all its technical replicates (on the right side of each image), and it is depicted with a different color (in grey LP02, in blue LP-CT, in green LP09 and in red LP01). On the left side of each image, the IR Biotyper clustering of the spectra into the corresponding isolates is shown, as well as the relationship between the different isolates (indicated by the branches of the dendrogram)
